# The Progress of Methylation Regulation in Gene Expression of Cervical Cancer

**DOI:** 10.1155/2018/8260652

**Published:** 2018-04-16

**Authors:** Chunyang Feng, Junxue Dong, Weiqin Chang, Manhua Cui, Tianmin Xu

**Affiliations:** The Second Hospital of Jilin University, Jilin, Changchun 130041, China

## Abstract

Cervical cancer is one of the most common gynecological tumors in females, which is closely related to high-rate HPV infection. Methylation alteration is a type of epigenetic decoration that regulates the expression of genes without changing the DNA sequence, and it is essential for the progression of cervical cancer in pathogenesis while reflecting the prognosis and therapeutic sensitivity in clinical practice. Hydroxymethylation has been discovered in recent years, thus making 5-hmC, the more stable marker, attract more attention in the field of methylation research. As markers of methylation, 5-hmC and 5-mC together with 5-foC and 5-caC draw the outline of the reversible cycle, and 6-mA takes part in the methylation of RNA, especially mRNA. Furthermore, methylation modification participates in ncRNA regulation and histone decoration. In this review, we focus on recent advances in the understanding of methylation regulation in the process of cervical cancer, as well as HPV and CIN, to identify the significant impact on the prospect of overcoming cervical cancer.

## 1. Introduction

Cervical cancer, which is one of the three most common gynecological tumors, has been the fourth leading cause of cancer-associated death among women worldwide, as well as becoming the second most commonly diagnosed cancer in developing countries. According to statistics, newly diagnosed cases and cervical cancer-associated deaths are approximately 520,000 and 260,000, respectively, every year, which affected youth trends more clearly [1]. It is widely recognized that persistent infection of high-risk-HPV (hr-HPV) accounts for the process from cervical intraepithelial neoplasia (CIN) to neoplasms, and vaccines of HPV and application of screening methods contribute a lot towards cervical carcinoma prevention. However, for established infections, vaccines have limited function and full-type coverage has not been achieved yet [1]. Additionally, as the 5-year survival rate is about 15% among advanced patients, the prognosis still remains unoptimistic in the late stages [2, 3]. Hence, it cries out for investigating the underlying molecular mechanisms on different biological expression levels to understand the genesis and progression of cervical cancer.

While gene mutation theory is incapable of providing reasonable explanations for many biological changes in tumor development, epigenetic alteration is drawing more attention, which involves modifications such as methylations of DNA and RNA, acetylations of histone, and regulations of ncRNA and aberrant chromatin. Methylated modification is extensively studied these years. DNA methylation mainly occurs at CpG islands where the methyltransferase DNMT family mediates the transfer of a methyl group to cytosines, generating 5-methylcytosine (5-mC), which can be oxidized into 5-hydroxymethylcytosine (5-hmC), 5-foC, and 5-caC by TET proteins step by step, so that methylation is achieved reversibly [4, 5]. Methylation decoration in RNAs is as common as it is in DNAs. M6A is one of the markers in mRNA methylation, and modifications take place in nascent pre-mRNAs predominantly [6]. Additionally, miR-RNAs and lnc-RNAs take part in epigenetic modifications themselves, and their biological functions are affected by the methylation state at the same time.

In this article, we summarize several recent studies of methylation regulation in the field of cervical cancer and discuss the potential of these molecular mechanisms in the period of gene expression, to get some enlightenment in epigenetics to carry forward the prevention and treatment of cervical cancer.

## 2. Hydroxymethylation and Cervical Cancer

### 2.1. Hydroxymethylation and Its Regulations

In 1972, 5-hmC was initially found in bacteriophages and then in mammalian DNA. Currently, 5-hmC, a more stable epigenetic mark than 5-mC, plays an important role in epigenetics and works as an intermediate in demethylation [7]. It has been confirmed that the brain has the highest concentration of 5-hmC, while the rectum, liver, colon, and kidney are subordinate. In contrast, 5-hmC is at a low level in the lung, placenta, and breast [8]. The regulation of DNA hydroxymethylation is mediated by several factors, among which human ten-eleven translocation (TET) is identified as a dioxygenase for converting 5-mC to 5-hmC; meanwhile, *α*KG, Fe^2+^, and ascorbate may activate the TET proteins as cofactors [9].

The TET protein family consists of TET1, TET2, and TET3, and their C-terminal catalytic domains come from a high degree of homology, which can be regulated by CXXC finger protein 1 (CFP1). Different CXXC domains have different functions; the CXXC5 domain of TET2 is able to downregulate TET2 with a 5-hmC decrease. But CXXC4 was found to be binding to the unmethylated DNA of TET1, TET2, and TET3, which then starts a caspase-dependent degradative process [10]. Some researchers found that in TET1-lacking cells, 5-hmC was reduced while 5-mC was increased. Moreover, TET1 can control 5-hmC by regulating hydroxylase activity to convert 5-mC to 5-hmC, which is HIF-1 dependent; at the same time, TET1 can also bind to CpG regions to stop some DNA methyltransferase activity [11]. It was demonstrated that TET3 is important for proper DNA repair, cell survival, and promotion of 5-hmC [12]. Besides, 5-hmC levels are also partly regulated by microRNAs. There are also some genes regulating 5-hmC, such as IDH1, IDH2, SDH, and FH [13]. Those factors are linked to the alteration of 5-hmC levels in cancer.

### 2.2. DNA Hydroxymethylation in Cervical Cancer and Other Cancers

To have cervical cancer treated and diagnosed precisely, many researches about 5-mC and other epigenetic modifications of cervical cancer aim to find treatment methods and diagnostic markers. But 5-hmC of cervical cancer is less researched, as there are only two articles about 5-hmC in cervical cancer.

Zhang et al. used immunohistochemistry to detect the expression of 5-hmC, 5-mC, and TET1/2/3 in 140 cervical squamous cell carcinoma (CSCC) tissues and 40 normal cervical tissues. They found that the expression of 5-hmC was an independent prognostic factor of squamous cell carcinoma, and compared with normal cervix tissues, the level of 5-mC was increased while 5-hmC was significantly decreased, which predicts poor prognosis of CSCC. Moreover, only the expression of TET2 was decreased in CSCC [14]. In contrast, Bhat et al. found that the 5-mC and 5-hmC levels were both significantly reduced in squamous cell carcinoma, but receiver operating characteristic curve analysis showed a significant difference in 5-mC and 5-hmC between normal and squamous cell carcinoma tissues. They also tested the promoter methylation of 33 genes; only PROX1, NNAT, ARHGAP6, HAND2, NKX2-2, PCDH10, DAPK1, RAB6C, and PITX2 could effectively tell the difference among the various stages of tumor with high sensitivity and specificity [15]. Expressions of 5-hmC and 5-mC in cervical cancer need further demonstrations, and these related results may serve as useful biomarkers for the early detection and accurate management of cervical cancer.

Although 5-hmC was studied little in cervical cancer, it is a noticeable part in other cancers; scientists have been making further studies for deeper mechanisms of 5-hmC as well.

It is demonstrated that TET1 and TET3 catalyze the conversion from 5-mC to 5-hmC by activating the TNF*α*-p38-MAPK signaling axis and inducing tumor malignancy and poor prognosis in breast cancer patients [16]. In prostate cancer, the androgen receptor decreases the expression of miR-29b which targets both TET2 and 5-hmC; 5-hmC represses FOXA1 activity, while its reduction activates the mTOR pathway and AR of prostate cancer [17]. In DLD1 cells, knockdown of TET1 will promote cancer cell growth, migration, invasion, and even epithelial-mesenchymal transition (EMT) which can also reduce UTX-1 but increase the EZH2 expression which can cause a loss of H3K27 methylation at the epithelial gene E-cadherin promoter [18]. In contrast, the levels of TETs are similar in colorectal tumor tissue and normal tissues. TET2 targets promoters marked by 5-hmC in normal tissue and turns it to colorectal cancer tissue [19].

## 3. DNA Methylation in Cervical Cancer and CIN

In cervical lesions, aberrant DNA methylation includes hypomethylation and hypermethylation. In cervical cancer and high-grade cervical intraepithelial neoplasia, most genes are hypermethylated; only three promoter regions are hypomethylated (Table 1).

### 3.1. Gene Hypomethylation in Cervical Cancer and CIN

Hypomethylation often occurs in the promoter region of genes, regardless if the gene is for a protein or RNA. The STK31 gene targets at oncogene E7 of HPV16. Its promoter/exon 1 is hypomethylated in HPV16/18-positive cervical cell lines, which induces an integration of HPV16E7/E6 [20]. The COL17A1 promoter is also hypomethylated in cervical cancer, and it precisely predicts both the increased invasive nature and patient outcome [21]. In CIN tissues, the rDNA promoter region reveals significant hypomethylation at cytosines in the context of CpG dinucleotides, which can result in an increase in rRNA synthesis in the development of human cervical cancer [22].

### 3.2. Gene Hypermethylation in Cervical Cancer and CIN

#### 3.2.1. Genome-Wide Studies of Aberrant Gene Methylation

There are some genome-wide studies of aberrant gene expression and methylation profiles which reveal susceptibility genes and underlying mechanisms of cervical cancer. In one study, a total of 1357 DEGs as well as 666 cervical cancer- (CC-) related methylation sites were screened out and 26 DEGs with 35 CC-related methylation sites were identified; ACOX3, CYP39A1, and DPYS are potential risk markers in CC, which were significantly enriched in 25 subpathways of 6 major pathways. EDN3 and EDNRB might play important roles in the molecular mechanism of CC [23]. In another study, 32 genes that might be associated with prognosis in the stages between Ib1 and IIa cervical cancer are profiled, among which the VIM gene is frequently methylated in CSCC and VIM methylation might predict a favorable prognosis [24]. The 14 hypermethylated genes, including ADRA1D, AJAP1, COL6A2, EDN3, EPO, HS3ST2, MAGI2, POU4F3, PTGDR, SOX8, SOX17, ST6GAL2, SYT9, and ZNF614, are implicated in *β*-catenin signaling in cervical carcinogenesis [25].

#### 3.2.2. Gene Hypermethylation Found in Cervical Cancer/CIN Tissue Cell Lines and Patients' Plasmas

Gene hypermethylation is found in CIN cervical cancer tissues, cervical cancer cells, and even cervical cancer patients' plasmas. The methylation rates of IFN-*γ*, FHIT, MGMT, CDKN2A, SALL3, and gene promoters were significantly higher in cervical cancer tissues than those in CIN and normal cervical tissues, which are related to the progression of cervical oncogenesis. CDKN2A methylation may lead to the development of malignant disease by increased p16(INK4A)/p14(ARF) expression [26–28]. LINE-1, HS3ST2, CCNA1, EPB41L3, EDNRB, LMX1, and DPYS were hypermethylated in cervical cancer tissues, CIN III and CIN II, versus normal tissues and CIN I, of which EPB41L3 seems to be the best marker. CADM1 is regulated by p53, and CADM1/MAL is hypermethylated in the HPV16/18-infected cell lines. The methylation status in cervical scrapes appears to represent the worst underlying lesion, particularly CIN III and cervical cancer. Results imply that hypermethylation of these genes may be highly associated with the development of cervical cancer [29–31]. Specific hypermethylated genes serve as the early prevention and prognostic prediction for cervical cancer. The different methylation statuses of all three genes PAX1, SOX1, and ZNF582 showed reasonable concordance in normal control samples as well as CIN I, CIN II, CIN III, and SCC samples [32, 33]. The promoter methylation statuses of DAPK1, MGMT, and RARB were positively correlated with the cervical disease grades, respectively. DAPK1 combined with the other two showed a significantly positive correlation with cervical disease grade as well [34]. The promoter hypermethylation of Keap1 significantly increased nuclear NRF2 expression in cervical cancer tissues, which is a marker of poor prognosis in patients with cervical cancer [35]. And the promoter of GPX3 is significantly downregulated due to its promoter hypermethylation in cervical cancer tissues; at the same time, GPX3 expression plays a role in the development of cervical squamous cell carcinoma and is significantly related to lymph node metastasis and prognosis in cervical cancer patients [36]. Promoter methylation and the loss of LDOC1 expression are frequent events in cervical cancer and could be potential molecular markers in cervical cancer [37]. Hypermethylation of RASSF2A and TSLC1 downregulating the expression of RASSF2A and TSLC1 was detected, which predicts a greater risk of progressing towards invasive cervical cancer [38, 39]. Hypermethylation of DOC2B promotes colony formation and cell proliferation, induces cell cycle arrest, and represses cell migration and invasion deeply; the promoter region of the DOC2B gene inhibiting AKT1 and ERK1/2 signaling is hypermethylated in premalignant and malignant cervical tissues and cervical cancer cell lines [40]. Those gene promoter methylations may be correlated with clinical stage and tumor grade and play a crucial role in cervical cancer progression.

The level of MEG3 methylation is significantly higher in cervical cancer tissues and patients' plasmas than in adjacent normal tissues and plasmas of healthy participants, respectively [41]. Promoter hypermethylation of some other genes like MYOD1, CALCA, hTERT, and RASSF1A can also be detected in serum samples of cervical cancer patients and are related to lymph node metastasis and FIGO stage [42, 43]. In conclusion, the present studies clearly showed that MEG3, MYOD1, CALCA, hTERT, and RASSF1A methylation in plasma can serve as diagnostic and prognostic biomarkers for cervical cancer patients, providing useful information for clinical management.

#### 3.2.3. Gene Hypermethylation Found in Different Ethnicities

The hypermethylation status of genes in cervical cancer patients is associated with different countries. In the North Indian population, methylation of the p16 gene promoter which induces loss of tumor-suppressing activity and promotes the development of cervical cancer is observed significantly in FIGO stage III [44, 45]. Meanwhile, correlated with clinical parameters, promoter hypermethylation and expression loss of PARK-2, RAR*β*, and FHIT are significantly higher in cervical cancer than in CINs and normal tissues, resulting in a significant association with tumor stage and histological grade [46, 47]. In Uighur women, increased methylation was detected at 13 CpG sites, and a high methylation level was associated with the risk of CIN2^+^; the strongest related site was 6650 [48]. The methylation level of the ERp57 gene promoter is higher in CSCC than in CIN, and normal tissues in Uighur women. Hypermethylation occurs only in certain CpG islands and sites, such as CpG1, CpG5, and CpG7, and it differs significantly in CSCC, CIN, or control groups [49]. In Uygur and Han, aberrant methylation of TFPI2 is present in a higher proportion of invasive cervical carcinoma (ICC) clinical samples [50]. Apart from that, hypermethylation is related to different age groups as well. Hypermethylation of the CDKN2A gene promoter is a frequent epigenetic change in younger patients with cervical carcinoma and implies a significant epigenetic role in tumor development in this age group [51].

### 3.3. The Relationship between HPV and Aberrant DNA Methylation in Cervical Cancer/CIN (Figure 1)

On the one hand, HPV and aberrant host gene methylation contribute to CIN and CCA, respectively, methylation of HPV can prevent itself from cleaning to keep the persistent infection state, and the host methylation level can also reflect the level of HPV-associated CCA. On the other hand, the HPV genome and host act on each other by methylated regulation. HPV takes part in the methylation of host genomes such as FAM19A4 and LHX1; the methylation of HPV itself can also work with the methylation of PAX1 and SOX1 in the host to enhance transcription, both of which induce bad outcomes of the host cervix.

#### 3.3.1. Methylation Status of HPV Genome in Cervical Cancer/CIN

HPV genome epigenetic alterations play an important role in cervical cancer progression. Among them, methylation of CpG sites in the L1, L2, and LCR regions in different types of HPV is studied most, and several deep relationships between the methylation of those regions and cervical cancer/CIN have been found out. HPV L1 gene methylation was the risk factor to cervical and elevated levels. HPV16 L1 methylation affects E6/E7 mRNA levels and can detect high-grade cervical lesions (CIN2^+^) [52, 53]. It also prolongs the cleaning of HPV infection and increases the risk of HPV cleaning failure in premalignant cervical lesion patients [54]. Besides, a panel of 12 HPV16 CpG sites which are methylated in L1, L2, and E5 can work as an informative biomarker for the triage of women positive for HPV16 infection and is correlated with the severity of cervical neoplasia, even cervical cancer [55]. But some other evidence shows HPV16 L1/L2 DNA methylation weakly associated with cervical disease grade in young women, which means HPV DNA methylation as a biomarker must take into account women's age [56]. The L1 and L2 regions of other types of HPVs are methylated in cervical cancer/CIN. Aberrant methylation of CpG sites in the L1 and L2 regions of HPV18 and other high-risk HPV types including HPV31, HPV33, HPV45, HPV52, HPV51, and HPV58 relates with the progression from early-stage CINs and may be considered as a biomarker of the progression of cervical neoplasia [57, 58]. Another research shows that the methylation of L1 in HPV16, HPV18, and HPV52 does not only play an important role in cervical cancer alone. The methylation of most HPV types except HPV52 also works together with the methylation of host genes including PAX1 and SOX1, which leads to a more significant result of cervical cancer/CIN [59]. Combining HPV methylation with PAX1 methylation improves the clustering for CIN2^+^ and methylated CpG sites in HPV31 LCR, including position 7479 and/or 7485, which is the promoter distal E2-binding site, suggesting a potential regulatory mechanism for papillomavirus transcription [60].

#### 3.3.2. The Interaction between HPV and Aberrant Methylation of Other Genes

In cervical cancer/CIN, methylated HPVs and other genes correlate with each other and serve as diagnostic and prognostic biomarkers for cervical cancer. Methylated HS3ST2, CCNA1, EGFR promoter, FHIT, TFPI2, CpG6, and CpG15 sites were associated with HPV16 infection in the progression of cervical cancer [61–63]. The results indicate that methylated genes may play important roles and be effective targets for the prevention and treatment of cervical cancer. HPV infection is also associated with hypermethylation of the promoter region of SALL3, DLX4, and SIM1 genes, which should be a significant progression marker for HPV infection in cervical cancer [64]. The methylation-mediated gene silencing of PRDM14, a regulator of NOXA and PUMA-mediated apoptosis, becomes an important factor in the development of hr-HPV-ICC (invasive cervical carcinoma) and offers a novel therapeutic target for HPV-induced cervical cancers [65]. In addition, that FAM19A4 promoter methylation even altered DNA methylation seems to be associated with HPV infection and high-risk types of HPV-induced carcinogenesis in the uterine cervix, CIN3^+^, and may increase with disease progression [66]. Moreover, some of the methylated genes have been demonstrated as attractive markers for hr-HPV-positive women, with a high reassurance for the detection of cervical carcinoma and advanced CIN2/3 lesions, such as EPB41L3 and FAM19A4 [29, 67].

Not only can gene methylation affect HPV infection, but HPV also results in other genes' aberrant methylation. HPV can result in novel DNA methylation events, including FAM19A4, LHX1, NKX2–8, PHACTR3, and PRDM14 genes in cervical carcinogenesis [68]. Numerous pieces of evidence suggest that HPV16 E7 oncoprotein mediates DNA hypermethylation in the CCNA1 and CXCL14 promoter and suppresses gene expression. The data also shows that E7 induces CCNA1 methylation by forming a complex with Dnmt1 at the CCNA1 promoter [69, 70]. The potential carcinogenic mechanism of HPVs, including influencing the DNA methylation pathway to affect DNA methylation and mRNA expression levels of those genes, can be utilized not only as a biomarker for early detection, disease progression, diagnosis, and prognosis of cervical cancer but also to design effective therapeutic strategies.

#### 3.3.3. Identification of Cervical Cancer by HPV and Gene Methylation Test

Currently, the HPV DNA test is one of the most vital tools to identify the risk of cervical cancer/CIN. Some studies show that detecting the methylation status of a few kinds of genes can also give evidence for diagnosing CIN2^+^ or help the HPV test to improve the specificity and sensitivity in the detection of cervical cancer/CIN.

In an independent cohort test, the methylated PCDHA4 and PCDHA13 test is equally sensitive but more specific than the human papillomavirus (HPV) test in the diagnosis of CIN2^+^ [71]. Combining the triage by MAL/miR-124-2 methylation analysis with threshold-80 and HPV16/18 genotyping can reach higher CIN3^+^ sensitivity and identify women at the highest risk of cervical (pre)cancer [72, 73]. Combining parallel testing of PAX1, DAPK1, RARB, WIF1, and SLIT2 DNA methylation and HPV DNA increases specificity to identify cervical cancer and achieves better precision than single HPV DNA testing does [74–76]. Above all, methylation of some genes has a prospect to be an auxiliary biomarker for cervical cancer screening.

Now, cervical (pre)cancer is usually classified by histologic pathology, but cervical conization will lead to a high risk of premature delivery and abortion for patients. A quantitative measurement of HPV-type 16 L1/L2 DNA methylation has demonstrated its correlation with cervical disease grade. The best separation between normal and dyskaryotic samples is achieved by assessment of the L1/L2 CpGs at nucleotide positions 5600 and 5609 [77]. At the same time, CCNA1 promoter methylation serves as a potential marker for distinguishing between histologic LSIL (low-grade squamous intraepithelial lesion)/negative and HSIL (high-grade squamous intraepithelial lesion)/positive [78].

## 4. Methylation-Related Regulations on Other Levels in Cervical Cancer/CIN

According to the central dogma of molecular biology, epigenetic modifications also occur in the process of genetic information expression, such as the DNA level mentioned above, RNA level including mRNA and ncRNA (noncoding RNA, miR-RNA, and lnc-RNA are included), and protein level involving common protein or histone.

### 4.1. Pervasive Gene Expression Adjustment of Cervical Cancer/CIN at RNA Level

#### 4.1.1. m6A Induces Methylated Regulation in mRNA

mRNAs carry genetic information by encoding polypeptides or proteins; that m6A methylates mRNA is widespread in eukaryotic cells. N^6^-Methyladenosine (m6A), which is an abundant and conservative RNA modification, is involved in a series of biological processes such as differentiation, metabolism, immune tolerance, and neuronal signaling by impacting on mRNA splicing, export, localization, translation, and stability [79]. As the UV cross-linking immunoprecipitation and single-nucleotide resolution show, the distribution of m6A is not random in mature transcripts but concentrates around the 3′ untranslated regions (UTRs), stop codons, and is within internal long exons [80]. The reversibility of m6A is accomplished by the orchestrated action of a battery of enzymes or proteins: as readers, proteins YTHDF and hnRNP recognize m6A-containing mRNA; as writers, METTL3, METTL14, and the WTAP complex support RNA methylation; and as erasers, FTO and ALKBH5 prop up RNA demethylation [79, 81].

As investigations about relationships between various tumors and m6A deepen, some crucial targets of tumor biological processes are found. Theories of m6A are elucidated increasingly in GSC, AML, HCC, BRC, and so on. Inhibition of FTO not only suppresses growth and self-renewal but also prolongs the lifespan of grafted mice and restrains tumor progression additionally compared with overexpression of METTL3 [82]. However, research of m6A about cervical cancer is rarely covered.

#### 4.1.2. ncRNAs Play an Important Role in Methylation Regulation

As genomics analysis shows, there are numerous transcripts being generated in the human body; just 1–2% transcripts own the function of encoding polypeptides or proteins, and the remaining 98% noncoding products play vital roles in many biological process, including proliferation, differentiation, and apoptosis [83]. They are all hot topics in the field of apparent genetics.

With NGS and qRT-PCR applied, levels of various miR-RNAs in cervical cancer are evaluated: most miR-RNAs are downregulated and relevant downstream signal pathways or target genes and proteins are reported, such as SOX2 of miR-145, TCF of miR-212, Bcl-2 of miR-187, and NF-*κ*b of miR-429, performing significant relationships with FIGO stage, lymph node metastasis and prognosis of patients in clinic, and colony formation, tumor size, proliferation, differentiation, apoptosis, and invasion on the lab research *in vivo* and *in vitro* [84–87]. There are still some miR-RNAs upregulated in CCA, such as miR-9 [88]. However, Zhang et al. reported that miR-9 is downregulated in cervical cancer on account of hypermethylation of miR-9 precursor promoters, which weakens the inhibiting effect on activity of the IL-6/Jak/STAT3 pathway [89]. These different outcomes may be induced by the potentially different methylation status in the objects.

Impacts of miR-RNA on the progress from HPV infection to cervical cancer are nonnegligible. Morel et al. reported that miR-375 could destabilize HPV16 early viral mRNA and contribute to the regulation of E6/E7 expression, which indicated the role of miR-RNA in high-risk HPV-associated carcinogenesis [90]. Yeung et al. revealed that HPV16 E6 takes part in epigenetic regulation of host gene-associated cervical cancer development; HPV16 E6 methylates the promoter region of the host gene of miR-23b, C9, or f3; and downregulated miR-23b enhances c-MET pathway-induced apoptosis of cervical cancer cells [91].

lnc-RNA interacting with miR-RNA regulates cervical cancer biological activity. lnc-RNA MEG3 is negatively relevant with FIGO stages, tumor size, lymphatic metastasis, and HR-HPV infection, and downexpressed MEG3 in cervical cancer reduces the inhibition effect on miR-21-5p expression, which leads to less apoptosis and more proliferation of cancer cells [92]. There are some cases about interactions between lnc-RNA, miR-RNA, and histone. For example, Zhang et al. explained the regulatory mechanism of lnc-RNA PVT1, which is overexpressed in cervical cancer: PVT1 binds with EZH2 directly to activate EZH2 to increase the histone H3K27 trimethylation level of the miR-200b promoter so that downexpressed miR-200b enhanced proliferation, cycle progression, and migration [93].

### 4.2. Methylation Research Related to Cervical Cancer Therapy Applications

Many mechanisms of methylation-associated regulations become the targets of therapy in the fields of chemotherapy and radiotherapy. It has been shown that cisplatin as well as 5-azacytidine touch off cytotoxic and growth inhibitory effects *in vitro* by demethylating the promoters of ESR1, BRCA1, RASSF1A, MLH1, MYOD1, hTERT, and DAPK1 to reexpress these tumor-associated genes [94]. Narayan et al. identified inactivation of decoy receptors TNFRSF10C and TNFRSF10D as major target genes at the 8p MDR region. On the one hand, the promoter hypermethylation of TNFRSF10C was an early event in cervical tumorigenesis; on the other hand, inactivation of decoy receptors induced extrinsic-apoptotic-pathway-dependent cell death in the cooperation of TRAIL and cisplatin in the presence of DNA-damaging drugs [95]. These covers above demonstrate that methylation-associated regulation offers an idea for developing new therapy targets.

Besides, methylation modifications impact on the sensitivity of chemotherapy and radiotherapy. Radiosensitization occurs when SiHa cells accept the therapeutic regimen combining DNA methylation inhibitor hydralazine with a histone deacetylase inhibitor valproic acid; unexpectedly, the efficacy of cisplatin chemoradiation was increased under the use of two epigenetic drugs [96]. Furthermore, epigenetic modifications also participate in therapeutic resistance. A univariate and hierarchical cluster analysis uncovered that standard chemoradiation resistance contacts closely with lower ESR1 transcript levels as well as unmethylated ESR1, unmethylated MYOD1, and methylated hTERT promoter [97]. In an article about the suppressor of cytokine signaling (SOCS) family and cervical cancer, Kim et al. found that DNA methylation contributed to SOCS1 downregulation, and histone deacetylation may be the mechanism of SOCS1 and SOCS3 regulation; in the meantime, ectopic expression of SOCS1 or SOCS3 could induce radioresistance of HeLa cells [98]. Similarly, a research about type-I ribosome-inactivating protein trichosanthin reported that Smac demethylation was subdued and Twist was upregulated in TCS-resistance cervical cells, which indicated that aberrant mitochondrial methylation may be partly the reason for drug resistance [99].

## 5. Conclusion

Cervical cancer is likely to be the first tumor which can receive idealized prevention and cure depending on the vital status of HPV in the pathological process. In spite of the astounding advances of screening plans and HPV vaccines, cervical cancer is still threatening the physical and psychological health of females with the absence of effective treatment, surveillance indexes, and fundamentally unclear molecular mechanisms. Over the past decades, methylation modification has been identified as having a significant role in the generation of cervical cancer. With the development of methylation-detecting techniques, there may be more convenient choices to explore it, not limited to cells and tissues, but techniques like liquid biopsy to advanced clinical transformation. We believe that it can not only enrich the markers for the early diagnosis and prognosis evaluation with other biomarkers to improve sensitivity and specificity in the clinic but also provide targets for exploiting new drugs as well as modifying the sensitivity in radiotherapy and chemotherapy for cervical cancer. However, there are still some items to be investigated deeply. Firstly, studies about the relationships between 5-hmC or 6mA and cervical cancer are rare, especially the aspect of HPV infection. Secondly, some researches find that methylation modification does not act itself but correlates with other epigenetic forms such as ncRNA regulation and histone decoration; therefore, the effective application of methylation relies on the simplification of key points. Additionally, from HPV infection via CIN to cervical cancer, relevant researches of the dynamic pathogenesis are inconsecutive. Moreover, it is recognized that methylation regulation is reversible, which is the unique advantage of therapy; only by enabling the reversibility controllable can we make full use of the characteristic. In conclusion, 5-hmC of hydroxymethylation, 5-mC of methylation, and 6-mA of RNA methylation are typical mechanisms of the methylation modification in gene expression; some ncRNA and histone regulations are involved in methylation in the meantime, and these investigations have profound instructive significance in the process of overcoming cervical cancer.

## Figures and Tables

**Figure 1:**
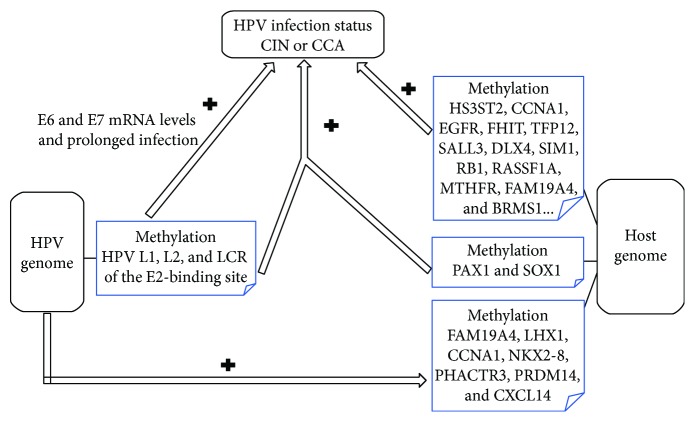
Methylation regulation between HPV and host genome in CIN or cervical cancer.

**Table 1:** DNA methylation of CIN or cervical cancer in recent studies.

Name of gene	Methylation status	Methylation-variable position	Function/relevant pathway	Reference	Notes
STK31	Hypomethylation	Promoter/exon 1	HPV oncogene-E6/E7	[20]	CIN III and CCA
COL17A1	Hypomethylation	Promoter	Collagen XVII	[21]	CCA
Ribosomal DNA	Hypomethylation	Promoter	rRNA synthesis	[22]	CIN II-III, CCA
EDN3 and EDNRB	Hypermethylation	Promoter	MAPK signal pathway MITF-Wnt/*β*-catenin signal pathway	[20, 23]	
VIM	Hypermethylation	Promoter	Epithelial-mesenchymal transition and aggressiveness	[24]	Ib1 and IIa stages of CCA
AJAP1 and SOX17	Hypermethylation	Promoter	Wnt signal pathway	[25]	
SFRP1 and SFRP4	Hypermethylation	Promoter	Wnt/*β*-catenin signal pathway	[25]	
CDKN2A	Hypermethylation	Downstream region	p16(INK4A)/p14(ARF)	[26]	CIN and CCA
IFN-*γ*	Hypermethylation	Promoter	IFN-*γ*-cancer immunoediting	[27]	CIN II-III and CCA
SALL3	Hypermethylation	Promoter	hrHPV-induced immortalization and malignant transformation	[28]	HPV-infected
EPB41L3	Hypermethylation	Promoter	DAL-1 protein	[29]	CIN II-III
CADM1/MAL	Hypermethylation	Unmentioned	Lesion-specific	[30]	CIN II-III and CCA
PAX1	Hypermethylation	Promoter	Unclear yet	[32]	CIN and CCA
DAPK1	Hypermethylation	Promoter	Epithelial-mesenchymal transition	[34]	CIN III and CCA
Keap1	Hypermethylation	Promoter	NRF2	[35]	CCA
GPX3	Hypermethylation	Promoter	Repair oxidative damages and lymph node metastasis	[36]	CCA
LDOC1	Hypermethylation	Promoter	Nuclear transcription factor	[37]	CCA
RASSF	Hypermethylation	Promoter	Ras protein	[38, 42]	CCA or plasma of CCA
DOC2B	Hypermethylation	Promoter	AKT1 and ERK1/2 signal pathway	[40]	CIN and CCA
MEG3	Hypermethylation	Promoter	Proliferation and apoptosis	[41]	Plasma of CIN III and CCA
